# Painful Finger

**Published:** 2014-09-08

**Authors:** Christopher Abraham, Leigh A. Price, Stephen M. Milner

**Affiliations:** Johns Hopkins Burn Center, The Johns Hopkins University School of Medicine, Baltimore, Md

**Keywords:** pain, nerve, neuroma, burn, amputation

## DESCRIPTION

**Figure 1:**
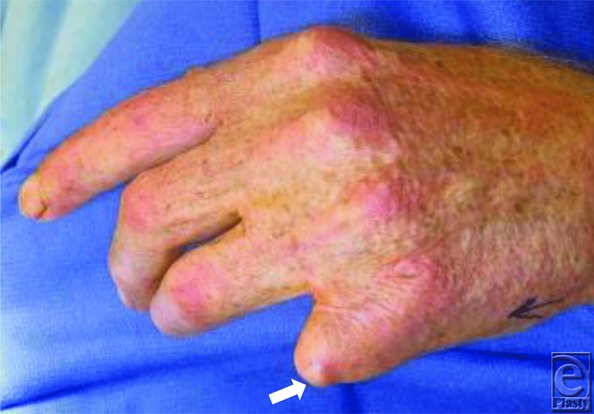
Left small finger amputation site with small round tender mass (white arrow) on ulnar aspect.

A 54-year-old man with an extensive history of burns to more than 60% of his body, some years ago, complained of a sharp, unremitting pain in the ulnar distribution of the left hand. Physical examination revealed a previously amputated left small finger with exquisite point tenderness and a small, 0.5-cm mass over its distal tip (Fig 1).

## QUESTIONS

1. **What is the diagnosis?**
2. **What is the differential diagnosis?**
3. **What is the pathophysiology behind its development?**
4. **What are the available treatment options?**

**Figure 2:**
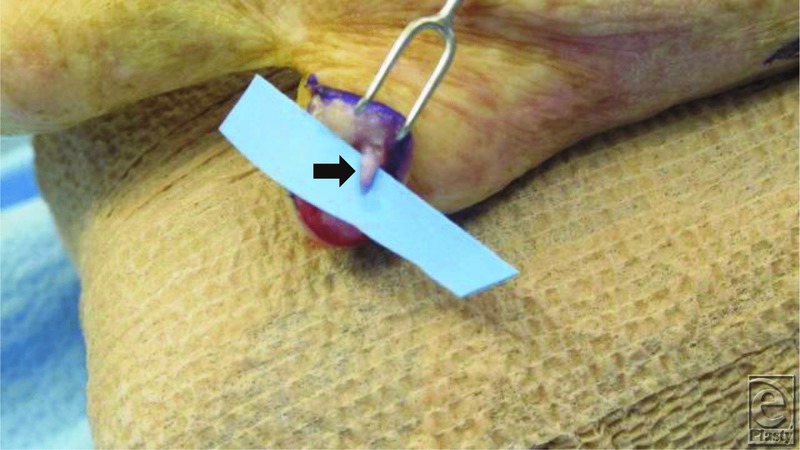
Illustrating neuroma (black arrow) of amputated site.

This patient has an amputation stump neuroma that formed at the end of a severed nerve. In this position, it was subject to repeated trauma leading to increased size, edema, fibrosis, and sensitivity. The pain was sufficient to limit the use of the entire hand. The diagnosis was easily made as tapping over the swelling elicited painful paresthesia radiating along the ulnar digital branch of the small finger (Fig 2).

The differential diagnosis includes traumatic neuroma, ganglion cyst, phantom pain syndrome, and chronic pain syndrome.^1^

After sharp trauma to an amputated peripheral nerve, fascicular escape and regenerating axons, lacking a protective endoneurial tube, grow into the surrounding scar. The result is a disorganized bulbous tumor that is often quite painful and tender with contact.^1^^-^3

Treatment of neuromas, especially those within the hand, is important due to their debilitating and painful symptoms, often altering patient productivity and activities of daily living.^2^ Multiple treatment options exist which focus on alleviating pain and restoring functional loss caused by the nerve injury.^3^ Wolfe et al has investigated methods which include relocation of the neuroma to an area of minimal contact, coagulation with electrocautery, silicone end capping, chemical sclerosis, corticosteroid injections, soft tissue coverage, and complete resection. Selecting the best treatment plan can be difficult because studies show conflicting results and postoperative axon regeneration is unpredictable. Thus no treatment regimen has been universally successful.^4^ In this patients' case, the decision was made to surgically explore the neuroma and allow the digital nerve to retract into the soft tissues.

## References

[1] Golan JD, Jacques L (2004). Nonneoplastic peripheral nerve tumors. Neurosurg Clin N Am.

[2] Vernadakis AJ, Koch H, Mackinnon SE (2003). Management of neuromas. Clinics Plast. Surg.

[3] Watson J, Gonzalez M, Romero A, Kerns J (2010). Neuromas of the hand and upper extremity. J Hand Surg.

[4] Wolfe SW, Hotchkiss RN, Pederson WC, Kozin SH (2010). Operative Hand Surgery.

